# World’s Columbian Dental Congress

**Published:** 1892-07

**Authors:** 


### World's Columbian Dental Congress.

The suggestion has already been made by one or more persons that the time of the meeting of the Columbian Dental Congress, in Chicago, would be a very favorable and proper time for holding re-unions by the alumni of the various dental colleges of our country, as doubtless every college of any importance will have a large number of its alumni present at that time. We can hardly conceive of anything that would afford more pleasure to the graduates of the various colleges of the country than to make arrangements for such re-unions. Let the alumni of every college inaugurate the movement; this could be done by the officers—president and secretary—of any and every alumni association, addressing circulars to all those who are graduates of the respective colleges, selecting one or two from each class, they to correspond and interest the members of their respective classes and make such arrangements as might seem best to them to secure an interesting and entertaining time. An opportunity would thus be afforded for the alumni of different classes of each and every college to become acquainted with one another, and to renew old acquaintances. Class histories might be presented that would be very interesting, indeed, many things could be done that would be entertaining and of real value.

The Dental Congress is to be held in a large hall, which has attached to it eighteen or twenty rooms, each of which would be large enough to accommodate from one to four hundred persons; and, we doubt not, arrangements could be made for the occupancy of these rooms for this purpose. Perhaps every college in the country has some of its alumni in Chicago who would act as local committees to make provision for the accommodation of their fellow graduates. The alumni of two or three colleges are now making arrangements for such re-unions, and it is very desirable that the sons of every college in the country should have the benefits of such a re-union. One day or a day and an evening could be well spent by such meetings.

In addition to these individual or college re-unions there could be a grand union meeting, for a day or an evening, of all the alumni of all colleges of the country. This, it is easy to conceive, might be made an exceedingly interesting occasion, indeed, these meetings could be made a very attractive feature of the Congress, though not a part of it nor under its guidance, they would nevertheless be interesting to all in attendance upon the Congress, and would, doubtless, embrace a large proportion of its membership. This much we will venture now, and, perhaps, will have something further upon the subject in the near future, after further consideration. We shall be glad to have an expression from all our brother editors throughout the country. Come, tell us what you think!

